# Temporal Change of SARS-CoV-2 in Clinical Specimens of COVID-19 Pneumonia Patients

**DOI:** 10.4269/ajtmh.20-0551

**Published:** 2020-07-08

**Authors:** Viravarn Luvira, Akanitt Jittmittraphap, Sant Muangnoicharoen, Nantarat Chantawat, Weena Janwitthayanan, Pornsawan Leaungwutiwong

**Affiliations:** 1 Department of Clinical Tropical Medicine, Faculty of Tropical Medicine, Mahidol University, Bangkok, Thailand;; 2 Department of Microbiology and Immunology, Faculty of Tropical Medicine, Mahidol University, Bangkok, Thailand;; 3 Tropical Medicine Diagnostic Reference Laboratory, Faculty of Tropical Medicine, Mahidol University, Bangkok, Thailand

## Abstract

The quality and type of specimen collection affect the sensitivity of real-time reverse transcriptase–PCR (rRT-PCR) for the diagnosis of SARS-CoV-2. In this report, the course over time of rRT-PCR for SARS-CoV-2 in 26 clinical specimens collected from the upper (nasopharyngeal and throat swabs) and lower (sputum) respiratory tracts of COVID-19 cases with pneumonia was investigated along with the clinical course. The preliminary results revealed that higher SARS-CoV-2 RNA concentration and longer time for detection make self-collected sputum a preferable specimen for the diagnosis and follow-up of COVID-19 pneumonia. Self-collection of sputum can minimize the risk of unnecessary exposure to healthcare workers, preserve the shortage of personal protective equipment, and limit viral transmission to the environment.

In December 2019, an outbreak of COVID-19, caused by SARS-CoV-2, began in China and had spread as a global pandemic.^1–3^ Real-time reverse transcriptase–PCR (rRT-PCR) has been the standard test for the diagnosis of SARS-CoV-2 in the early stages of the COVID-19 pandemic. However, the quality and the timing of specimen collection affect the sensitivity of the test.^4^ To date, no consensus practical guideline for specimen collection exists. Nasopharyngeal swabs (NPS) and throat swabs (TS) are widely used for the diagnosis of COVID-19. Detection of SARS-CoV-2 in different sample types and evaluation of the rRT-PCR results over time during the clinical course of the disease are limited so far. Herein, we describe temporal changes in rRT-PCR results for SARS-CoV-2 in respiratory-tract specimens, both NPS/TS and sputum, along with the clinical course of COVID-19 pneumonia.

We analyzed 26 clinical specimens from three cases of COVID-19 pneumonia hospitalized at the Hospital for Tropical Diseases in Bangkok during a COVID-19 outbreak in Thailand between March 2020 and May 2020. Pneumonia was diagnosed based on clinical symptoms and chest radiography. COVID-19 was confirmed by rRT-PCR for SARS-CoV-2 in different specimen types from the same day on clinical diagnosis and during the hospital course of pneumonia cases, including NPS/TS collected in a single aliquot of viral transport medium (VTM) and self-collected sputum in a container without VTM. The rRT-PCR for SARS-CoV-2 (detection kit for novel coronavirus 2019-nCoV RNA; DaAn Gene Co., Ltd., Guangdong, China) and respiratory pathogen panel for the detection and identification of 26 pathogens (Allplex™ Respiratory Panel Assays; Seegene Inc., Seoul, South Korea) were performed at the Tropical Medicine Diagnostic Reference Laboratory of the Faculty of Tropical Medicine, Mahidol University, Bangkok. A cycle threshold value (Ct value) less than or equal to 40 is defined as positive. The study was approved by the Ethics Committee of the Faculty of Tropical Medicine, Mahidol University, Thailand.

All COVID-19 pneumonia cases were admitted in isolation and were treated with a combination of favipiravir, chloroquine, and darunavir/ritonavir, or lopinavir/ritonavir according to the National Treatment Guidelines for COVID-19 at that particular time for at least 10 days.^5^ Antimicrobial agents were prescribed according to the physician’s decision. All cases were in isolation until the symptoms were clear and all clinical specimens revealed negative for SARS-CoV-2. The clinical and laboratory characteristics of the COVID-19 patients are summarized in Table 1.

**Table 1:** Clinical and laboratory characteristics of COVID-19 patients

	Case 1*	Case 2	Case 3
Age (years), gender	30, male	47, male	68, male
Probable source of infection	Local transmission, public transport	Local transmission, public transport	Imported, Pakistan
Preexisting medical illness	None	None	Type 2 diabetes mellitus, hypertension, and dyslipidemia
Symptoms and signs	Fever and myalgia	Fever, anosmia, crepitation right lung, and deoxygenation	Cough, wheezing both lungs, and deoxygenation
Chest radiography at diagnosis	Alveolar infiltration of the RLL field	Ground-glass infiltration at RLL	No pulmonary infiltration on admission
			Ground-glass infiltration at both lower lung fields at day 3 of admission
Diagnosis (day after onset)	3	8	3
rRT-PCR for respiratory pathogens	Negative	*H. influenzae* in both NPS/TS and sputum	*H. influenzae* in both NPS/TS and sputum
Duration from diagnosis to negative rRT-PCR for SARS-CoV-2 in NPS/TS vs. sputum (days)	14 vs. 18	12 vs. 12	27 vs. 40

rRT-PCR = realtime reverse transcription-polymerase chain reaction; *H. influenzae = Haemophilus influenzae*; NPS = nasopharyngeal swabs; RLL = right lower lung; TS = throat swabs.

* A full description of case 1 has been reported in ref. 13.

Interestingly, two of three COVID-19 cases were coinfected with *Haemophilus influenzae.* Coinfection with respiratory pathogens including bacteria, viruses, and fungus has been reported with the rate varying among the study population and method.^6–8^ However, the types of coinfected pathogens, the pathogen interaction, and disease severity among SARS-CoV-2–positive cases are unclear so far. Clinical and experimental studies to fill these clinical gaps are warranted.

The dynamics of SARS-CoV-2 RNA concentration in different types of clinical specimen from COVID-19 pneumonia patients during the clinical course are shown in Figure 1. The rRT-PCR technique was performed to detect the ORF1b and N-gene regions of SARS-CoV-2, both regions were well correlated in all specimens (Figure 1A and B). The revolution in clinical and rRT-PCR results of each patient was illustrated in Figure 1C–E. The overall trend of higher concentration of virus (indicated by lower Ct value) in sputum than NPS/TS from the same day was observed. Viral RNA could be detected for longer time in sputum (during > 2–6 weeks) than swab specimens in two of three cases, with an obviously longer time in one case (Figure 1E), implying that the correct types of specimens from the anatomical site of pathology—sputum in pneumonia cases—are preferable.^4^ Although some COVID-19 patients reported dry cough, expectoration can be achieved in all pneumonia cases without the induction of sputum. The higher positive rates of SARS-CoV-2 in sputum than TS on diagnosis have been recently reported; however, the data of dynamic changes of virus detection in sputum during the course of illness are limited.^9–11^ Importantly, the detection period of 2–5 weeks after the onset of symptoms (Figure 1C–E) in swab specimens in our study was longer than those reported recently in hospitalized COVID-19 patients, in which SARS-CoV-2 were positive in swabs mainly in the first week, but in sputum until the second and third weeks of illness.^12^ This discrepancy might be explained by the difference in severity of COVID-19 with moderate severity in our cases and mild cases in the previous study.^12^ Thus, these preliminary results suggested that sputum should be the specimen of choice for the diagnosis and follow-up of COVID-19 pneumonia. Self-collection of sputum is simple expectoration, less invasive, and minimizes the risk of transmission to healthcare workers, especially in settings with limited personal protective equipment.

**Figure 1.**
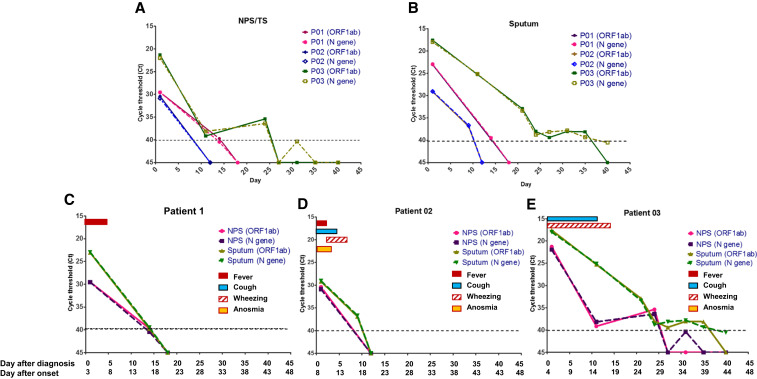
Detection results of respiratory specimens by real-time reverse transcriptase–PCR (rRT-PCR) during the clinical course of COVID-19 pneumonia. The concentrations of SARS-CoV RNA in clinical specimens are plotted by the cycle threshold value from nasopharyngeal and throat swabs (**A**) and sputum (**B**). The revolution in clinical and rRT-PCR results of the patients 1–3 (**C–E**).

Although the data from this relatively small sample size might limit the ultimate implications of this study, the strong relation between clinical and laboratory findings mirrors the unique feature of this report. However, virus isolation in cell cultures was not performed in this study, so it does not imply that the positive SARS-CoV-2 by rRT-PCR is still infectious.
